# P21 An assessment of knowledge and hand hygiene practices among healthcare workers at Dodoma Regional Referral hospital

**DOI:** 10.1093/jacamr/dlac004.020

**Published:** 2022-02-16

**Authors:** Erick Venant, Valence Ndesendo, Karin Wiedenmayer, Eva M. A. Ombaka

**Affiliations:** RBA Initiative, Dodoma, Tanzania; St John’s University of Tanzania, Dodoma, Tanzania; Swiss Tropical and Public Health Institute, Basel, Switzerland; University of Basel, Basel, Switzerland; St John’s University of Tanzania, Dodoma, Tanzania

## Abstract

**Introduction:**

The WHO-promoted ‘My 5 moments for hand hygiene’ is designed to prevent the spread of infectious organisms in healthcare settings which occurs mostly via contaminated hands of healthcare workers (HCWs), items/equipment and the environment. Information on its implementation in Tanzania is needed.

**Objectives:**

The main objective of the study was to assess knowledge, availability, and access to facilities for hand hygiene and the adherence to the WHO's five moments of hand hygiene by healthcare workers at Dodoma Regional Referral Hospital. The bacterial content of cell phones of HCWs was assessed as a source of microbial contamination.

**Methods:**

This was a descriptive cross-sectional study. Three sets of data were collected through (i) questionnaires to assess knowledge, attitudes, and barriers to effective hand hygiene; (ii) observation to assess the adherence of five moments of hand hygiene; and (iii) laboratory examination of cell phones for bacterial contamination. Analysis used Statistical Package for Social Science (SPSS).

**Results:**

Over 75% of HCWs had formal training on hand hygiene and had access to handwashing facilities, but only 63% were aware of the WHO five moments. Of 270 doctors, nurses, and healthcare students only 7 (2.6%) complied with expected action with no difference between groups. No hand hygiene was observed after touching cell phones. Thirty-four percent of sampled cell phones were contaminated with staphylococci species, which were resistant to penicillin and had varying resistance to erythromycin, clindamycin and gentamicin.

**Conclusions:**

Hand hygiene is the most effective way to reduce the risk of healthcare-associated infections. However, many healthcare workers do not adhere to recommended hand hygiene despite training and the availability of handwashing facilities. The low practice of hand hygiene means that cell phones of healthcare workers can easily act as a reservoir of transmissible organisms. The use of cell phones as a source of contamination must be included in the interventional training of HCW.

**Table 1.** Practice of WHO five moments of hand hygiene as observed in two wards (gynaecology & obstetrics and male orthopaedic ward)

Total no. of opportunities	HH action before touching the patient		HH action after touching the surroundings exemplified by mobile phones		HH action before aseptic procedure	
269	Yes	6	Yes	0	Yes	1
	No	157	No	41	No	64
	163		41		65	
	The overall hand hygiene compliance was 2.6%					

**Figure 1.**
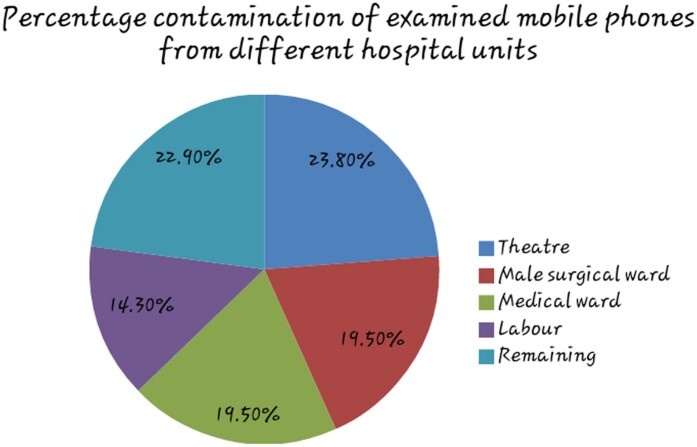
The results above show the highest contamination in the most sensitive wards like theatre and male surgical wards.

**Figure 2.**
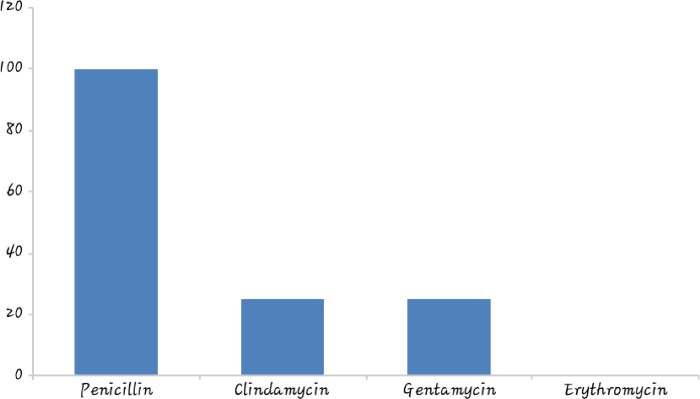
Percentage resistance of three tested samples from different three hospital units. From the results above the isolated bacteria obtained from the healthcare workers cell phones are showing resistant to commonly used antibiotics.

